# Moritz Schiff (1823–1896): A Physiologist in Exile

**DOI:** 10.5041/RMMJ.10064

**Published:** 2011-10-31

**Authors:** Moshe Feinsod

**Affiliations:** Faculty of Medicine, Technion–Israel Institute of Technology, Haifa, Israel

**Keywords:** Moritz Schiff, history of medicine, experimental physiology

## Abstract

Moritz Schiff was one of the pioneers of modern experimental physiology. His involvement in the liberal movement forced him out of Germany, and, because of his adherence to proper physiological research, he had to flee Italy, his first refuge. The number and importance of his contributions are outstanding. The aim of this paper is to raise interest in his biography and to present a yet unreported field of research that is regarded as the root of functional imaging of the brain.

He was “seeker after the truth” and a discoverer of it, and he was moreover a great teacher who could impart to others all he knew and even prepare them to follow his lead as to become discoverers themselves.^1^

## INTRODUCTION

Following the spread of the ideas of the French Revolution and their implementation by Napoleonic conquests, the first half of the nineteenth century saw, especially in the German states, the growing emancipation of the Jews. The struggle for equality was accompanied by an increasing number of young Jews seeking secular education in the gymnasia and universities, achieving high positions in culture, science, and medicine.

In the history of Medicine, the mid-nineteenth century is marked by the emergence of methodological experimental physiology, led by figures like Magendie in France and Müller in Germany who incorporated the achievements in physics and chemistry into physiology and employed instrumentation enabling the measurement and recording of the physiological data. The planned controlled experiment became the cornerstone of the budding scientific medicine.

These processes are personified by the eminent experimental physiologist, Moritz Schiff (1823–1896). His scientific achievements were hailed all over Europe and the US, but he was forced to live in exile, first because of his involvement in the German liberal movement and, later, because of his dedication to science clashing with prejudice and ignorance tainted by chauvinism.

In recent decades, new facets of his activities have been revealed. The aim of this paper is to contribute to the renewed interest by reporting on a rather ignored line of research that only nowadays is being appreciated as one of the roots of modern functional imaging of the brain.

## GERMANY

Moritz Schiff was born on January 28, 1823 to a prosperous Jewish merchant family in Frankfurt-am-Main. After matriculating from a German gymnasium and failing in commerce, he became an apprentice in the prestigious Schankenbergische Institute of Natural History and, in 1844, received his M.D. in Göttingen after studying physiology with the famous Johan Müller in Berlin. His love of the natural sciences took him to Paris where he studied under one of the founders of modern physiology, François Magendie (1783–1855), and with his pupils, François A. Longet (1811–1871) and Carlo Matteucci (1811–1868). Concomitantly, he worked at the Museum of Zoology in the famous Jardin des Plantes. In the summer of 1845, he returned to Frankfurt and, in 1846, obtained the position of the director of the ornithological part of the Institute where he had worked in his youth. Schiff classified the birds of South America and collaborated with Prince Charles Bonaparte, nephew of Napoleon I, who was a renowned authority in ornithology. In 1848, Schiff was swept by the liberal movement and joined the Baden army as a physician in a failed attempt to liberalize Germany. He was barely saved from execution and resumed his work at the Institute. The following years were very productive. Schiff studied the motor outflow from the brain and the microscopic anatomy of the nerves and their regeneration. He began to study the nervous influence on cardiac contraction and was the first to demonstrate, in 1850, the refractory period of the heart muscle. The innervations of the heart were a subject of numerous projects throughout his career. In another study he established the importance of the neck muscles on stability. His work was hailed, and the French Academy awarded him the most prestigious Monthyon Prize for his work on the influence of the autonomic nervous system on body temperature and bone nutrition.

At this stage Schiff applied for the position of *Privatdozent* in zoology at the University of Göttingen. The university was ready to accept him, but the Ministry in Hanover, taking intoaccount his ancestry and liberal revolutionary past, vetoed the appointment. In 1856, Schiff moved to Bern as an assistant professor of Comparative Zoology. He conducted studies on the influence of the autonomic nervous system on the production of sugar in the liver, thus explaining Claude Bernard’s observation of the appearance of diabetes following some brain lesions. It was during these studies that Schiff described the occurrence of extension of the forelimbs together with paradoxical respiration as a grave prognostic sign after spinal cord injury. This observation was repeated by Sherrington 40 years later, and the eponym “Schiff–Sherrington reflex” was thus coined.^2^

In 1856 Schiff demonstrated that animals of various species could not survive after removal of the thyroid gland, but neither physiologists nor physicians of that time were prepared for the study of this ductless gland. This pioneering work passed unnoticed, to be recognized only three decades later.^3^

## FLORENCE

After Italy’s second War of Independence, it was decided to restore the prestigious academic level of the Italian universities that had suffered during the post-Napoleonic Austrian occupation. Matteucci, the renowned physiologist and Schiff’s former mentor in Paris, was now an eminent statesman, and he invited Schiff, whose fame as a superb experimenter had spread, to chair and lead physiological research at the University of Florence. In 1862 Moritz Schiff went to Florence together with his brother Hugo, the inventor of the Schiff reagent.

**Figure 1:**
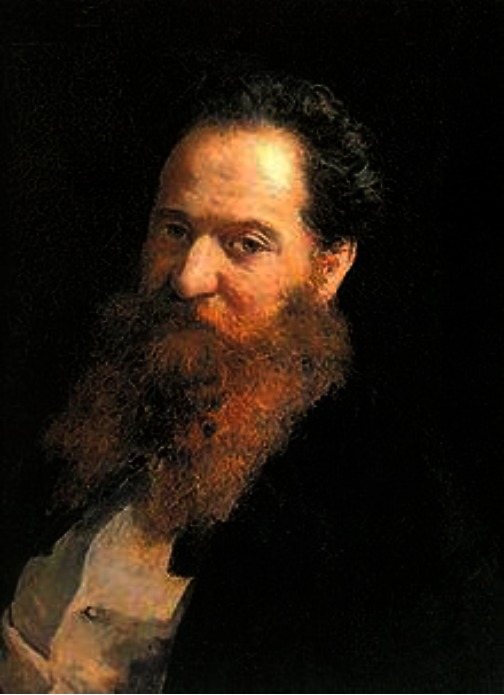
Moritz Schiff, circa 1860.

In Florence Schiff’s productivity flourished. He continued and expanded his studies on the vasomotor nerves and their central origin and on the innervations of the heart. As part of his neurophysiologic studies, he tried to quantify sensations by the size of the pupil (Figure 1).^4^

The production and fate of bile salts engaged many scientists. In 1860 Schiff proposed his solution to the problem. He returned the excreted bile, collected via a fistula, into the duodenum and demonstrated definitively the re-absorption of bile salts from the intestines in a positive feedback loop. In a much later experiment, he fed guinea-pigs ox bile salts and showed their presence in the excreted bile of the other species. The “Schiff biliary cycle” was thus established as an eponym (Figure 1).^5^,^6^

His fame as a leading experimentalist spread; many came from abroad to visit his laboratory. In 1874 T.G. Hake reported to the British medical community that, while conducting a study comparing ether to chloroform anesthesia, Schiff treated cardiac arrest caused by the latter by open chest cardiac massage and severe hypotension by rhythmic compression of the abdominal aorta. This treatment of cardiac arrest was readily adopted for laboratory animals and, at the turn of the century, for human patients, too.^7^

In 1869 Schiff published the results of a meticulous pioneering study in the emerging field of temperature changes in the nervous system evoked by sensory, motor, and psychic activity.^8^ The formulation of the first law of thermodynamics (“the conservation of force”) by Helmholtz in 1847 gave physiologists and physicians the theoretical basis and the impetus to study metabolic activity, expressed as temperature changes, in the organs of animals and humans in health and disease. A formidable challenge was to explore whether such changes occur in the nervous system. Schiff posed three questions to be researched (as expressed in present-day terms):

1. Is the stimulation of a sensory nerve transmitted to the cerebral hemispheres, or is it stopped at the brain-stem?
2. Does the propagation along the nerves depend on metabolic activity?
3. Is perception accompanied by temperature changes in the brain that are measurable with available instruments?

The instrument chosen by Schiff was the thermocouple needle which had been recently developed by Becquerel and Breschet^9^ and could be inserted with minimal injury into the brain or applied directly to exposed nerves of experimental animals. Schiff’s conclusions may be summarized into the following main points:

1. The stimulation of a nerve increases its temperature.
2. The stimulation of all the sensory modalities causes elevation of brain temperature.
3. The brain’s response to sensory stimulation is abolished by morphine.
4. Repeated stimulation of the sensory modalities is followed by diminishing brain response.
5. “Psychic excitation” set off by any sensory modality causes an elevation of brain temperature that is higher than by less complex sensations.

The data collected in Schiff’s experiments cannot match modern physiological requisites; galvanometers were still cumbersome, the readings were subjective, and no statistical tools were yet employed to ascertain the validity of the results. Despite these shortcomings, his conclusions were correct. They enabled Claude Bernard, who was fond of Schiff’s work, to declare: “The results of recent experiments do not leave room for doubt. Each time the spinal cord or a nerve exhibit sensitivity or movement, each time the brain performs intellectual work, a corresponding amount of heat is produced.”^10^ In the following years, several investigators, some being of Schiff’s own pupils, attempted to make a too long leap and measure temperature changes in the human cortex non-invasively. This ambitious leap ended in a crash, but the attempts initiated by Schiff are nowadays regarded as the very early roots of functional imaging of the brain.^11^

It is quite curious that this important and pioneering contribution of Schiff is missing from his former biographical notes and is reported here for the first time.

Soon after going to Florence, Schiff declared openly his attitude to vivisection in the popular daily journal, *La Nazione* (January 1864). He regarded the use of animals as a necessity permissible only when fulfilling two conditions: the research could be conducted only on a whole animal, and it should be rendered painless by using general anesthesia. For nearly 10 years he worked peacefully, but trouble started upon the arrival in Florence of the British activist, Frances P. Cobbe, who started a vicious crusade against the use of animals in Schiff’s experiments. In her campaign, Cobbe wrote defamatory letters to the British press that became increasingly tainted with anti-Semitic rhetoric, incited the British community of Florence, and, finally, some influential local figures added chauvinistic arguments to the vicious campaign. The *British Medical Journal*^12^ came out in defense of Schiff and condemned the malicious ignorance of the offenders, but in vain. Schiff fought back, but the subject became a court issue, and he had no choice but to flee to a new haven, Geneva.

## GENEVA

The Faculty of Medicine in Geneva was opened in 1872. Four years later, after the resignation of Brown-Séquard from a chair he never occupied, Schiff accepted an invitation to chair the Department of Experimental Physiology. After a prolonged public debate in which Schiff convinced the anti-vivisectionists that his experiments were necessary for the advancement of medicine and were conducted under impeccable ethical care, he was finally able to proceed with his experiments.

Schiff’s new laboratory became a center for visits by many scientists and was hailed by Claude Bernard as well as by German scientists. He became known as a scientist who took nothing for granted, one who checked and rechecked his results to perfection. It was no wonder that when two eminent Swiss surgeons, Theodor Kocher and Jacques-Louis Reverdin, struggled with goiters common in Switzerland, Schiff was asked to return to his thyroidectomy experiments of 1856. Schiff demonstrated again that the effects of thyroidectomy in humans were identical to those in all other mammals. His pioneering discovery was that thyroid “grafts into the peritoneum reversed, though temporarily, the effects of thyroidectomy”. He therefore suggested preparing “a thyroid paste” for repeated injections, but explained that his laboratory conditions were not suitable for such a project. It is plausible that this decision, stemming from a sense of responsibility, pushed Schiff’s achievement to near-oblivion and he was barely cited. Only in 1891 could George R. Murray report the successful treatment of a human patient by injections of thyroid extracts. The solid, well-grounded accomplishment and foresight of Schiff in thyroid research stand in contrast to the imprudent self-injections of testicular extracts by which Brown-Séquard won fame as the “father of organotherapy”.^13^–^16^

**Figure 2:**
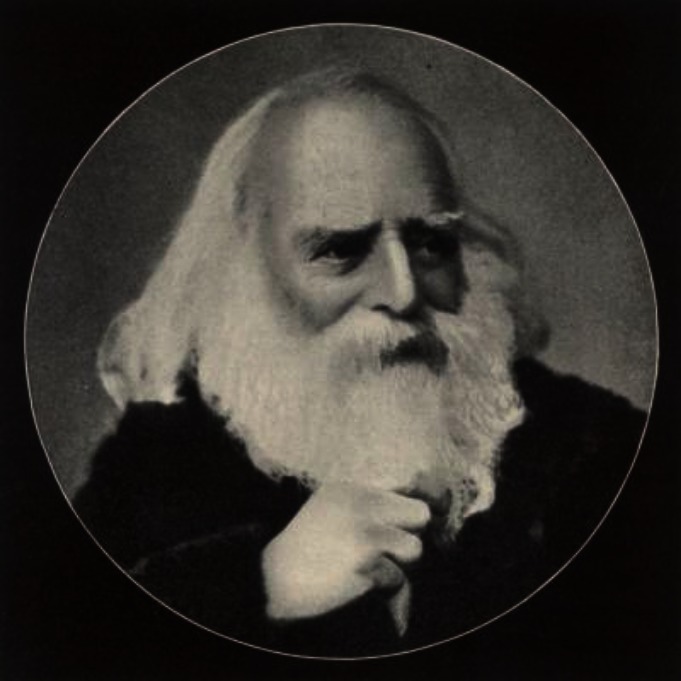
Moritz Schiff, circa 1890.

The liberal atmosphere in Geneva attracted many Jewish, Polish, and other ethnic groups, as well as women students and young scientists from the Russian Empire whose entry to their local universities was denied because of *numerus clausus*.^17^ Dr Hillel Yaffe (1864–1936), who became a pioneer physician in Northern Eretz–Israel, was one of those students, and in the years 1888–1889 he served as an assistant to Schiff. In an affectionate letter (in French) to H. Friedenwald, Yaffe portrayed his “beloved teacher” as a man of “very short stature, his beautiful face framed by a white beard and long hair, with piercing though kind gray eyes … modest to the extreme, negligent of his clothing, interested only in Science and in social deductions, without admitting the commonplace appropriateness and what we call the laws of Society”(Figure 2).^18^ Schiff was an indefatigable worker who professed that the best relaxation was to move to another subject. Indeed, his laboratory consisted of a very large hall with many laboratory benches at which groups of assistants performed various experiments.

An enigmatic sentence in Yaffe’s letter deserves special attention: “Many of the things that he had discovered, or that he had anticipated, were published under other names and he, even if with some resentment, accepted it philosophically without murmur and opened himself only to his assistants or scholars of his family.” It is tempting to read this enigmatic sentence as evidence that Schiff, during the height of the vivisection controversy, chose to publish under a pen name to avoid prosecution and persecution. If it were really so, he could, in the liberal atmosphere of Geneva, reclaim his authorship. Another possibility, entertained by the present author, is that Schiff refers to findings or ideas that were expropriated.

Yaffe reported that, despite not recognizing religion or nationality, Schiff always asserted his ancestry and sympathized and protected the young Jewish students with profound indignation for their prosecutors. In this spirit he secured the position of Assistant Professor of Physiology in 1888 for the exiled DrWaldemarMordechaiHaffkine that enabled him to go to Paris and London later, to build his career as the developer of the anti-vaccine for cholera and bubonic plague.^19^

Very little is known about Schiff’s personal life and family. He married his cousin, Claudia Trier. Their son, Roberto, became a professor of Chemistry at the University of Pisa. Mario, a son from a second marriage, became a professor of French Literature in Florence.

Several years prior to his death from diabetes in 1896, his adoring students and assistants started to collect his published scientific books as well as the text-books of physiology. The articles in German and French were assembled in a four-volume book entitled “*Moritz Schiff’s Gesammelte Beiträge Zur Physiologie”* (Lausanne, 1894–1898). In volume 1, Schiff himself rearranged some of his articles on centers in the nervous system that are related to respiration.

Immediately after his death, the *British Medical Journal* published a highly praising obituary but, in the last century, there were only a few attempts 1,5,13,18,20–22 to recognize Schiff’s contributions to nearly all fields of physiology, at a period when experimental physiology was still taking its formative steps. Schiff should also be regarded as a person who paid dearly for his adherence to the ideas of freedom and liberalism and to genuine physiological research. His personality, contributions, and impact deserve a thorough biography.
